# Parkinson’s disease multimodal imaging: F-DOPA PET, neuromelanin-sensitive and quantitative iron-sensitive MRI

**DOI:** 10.1038/s41531-021-00199-2

**Published:** 2021-07-08

**Authors:** Frédérique Depierreux, Eric Parmentier, Laurane Mackels, Katherine Baquero, Christian Degueldre, Evelyne Balteau, Eric Salmon, Christophe Phillips, Mohamed Ali Bahri, Pierre Maquet, Gaëtan Garraux

**Affiliations:** 1 GIGA-CRC In Vivo Imaging, University of Liège, Liège, Belgium; 2 Department of Neurology, University Hospital of Liège, Liège, Belgium; 3 GIGA In Silico Medicine, University of Liège, Liège, Belgium

**Keywords:** Parkinson's disease, Diagnostic markers

## Abstract

Parkinson’s disease (PD) is a neurodegenerative synucleinopathy characterized by the degeneration of neuromelanin (NM)-containing dopaminergic neurons and deposition of iron in the substantia nigra (SN). How regional NM loss and iron accumulation within specific areas of SN relate to nigro-striatal dysfunction needs to be clarified. We measured dopaminergic function in pre- and postcommissural putamen by [^18^F]DOPA PET in 23 Parkinson’s disease patients and 23 healthy control (HC) participants in whom NM content and iron load were assessed in medial and lateral SN, respectively, by NM-sensitive and quantitative R2* MRI. Data analysis consisted of voxelwise regressions testing the group effect and its interaction with NM or iron signals. In PD patients, R2* was selectively increased in left lateral SN as compared to healthy participants, suggesting a local accumulation of iron in Parkinson’s disease. By contrast, NM signal differed between PD and HC, without specific regional specificity within SN. Dopaminergic function in posterior putamen decreased as R2* increased in lateral SN, indicating that dopaminergic function impairment progresses with iron accumulation in the SN. Dopaminergic function was also positively correlated with NM signal in lateral SN, indicating that dopaminergic function impairment progresses with depigmentation in the SN. A complex relationship was detected between R2* in the lateral SN and NM signal in the medial SN. In conclusion, multimodal imaging reveals regionally specific relationships between iron accumulation and depigmentation within the SN of Parkinson’s disease and provides in vivo insights in its neuropathology.

## Introduction

Parkinson’s disease (PD) is the second most frequent neurodegenerative disorder worldwide and a growing public health issue given population aging^1^. Yet, our understanding of PD remains fragmentary. Although the neuropathological hallmark of PD brain consists of the accumulation of insoluble synuclein deposits in both the peripheral and central nervous systems^2–4^, a strong clinical emphasis has been put on the neuronal loss in dopaminergic neuromelanin (NM)-pigmented neurons of the substantia nigra (SN) compacta^5^. Indeed, the decline of dopaminergic nigro-striatal neurotransmission is considered as the core mechanism explaining motor symptoms of PD and the diagnosis of PD still mainly relies on the clinical observation of motor signs^6^. The latter only appear when 50–60% of dopaminergic neurons of SN are already lost^7^. Unfortunately, even in typical PD cases, the accurate diagnosis of PD remains challenging: only 74–84% diagnostic accuracy is achieved, depending on the practitioner’s expertise in movement disorders^8,9^. Therefore, there is a need for reliable in vivo diagnostic biomarkers. Measurement of striatal [^18^F]-DOPA uptake by positron emission tomography (PET) is still regarded as one of the most reliable tool for the in vivo diagnosis of PD because it directly probes the nigro-striate synthesis of dopamine^10–12^, and as such considered as a measure of dopamine terminal loss. [^18^F]DOPA uptake is decreased in the putamen in virtually all patients with PD, even in the early stage of the disease^12^. Nevertheless, the access to [^18^F]-DOPA PET is limited and the technique exposes patients to radiation hazards. More recently, MRI sequences allowed for the estimation of SN content in NM^13–16^ as well as brain iron levels which induce an increase in apparent transverse relaxation rates (R2^∗^)^17^. In PD, it is expected that R2* would increase in SN due to iron accumulation, whereas the signal of NM-sensitive MRI would decrease in SN, due to dopaminergic neuron loss and depigmentation.

**Fig. 1:**
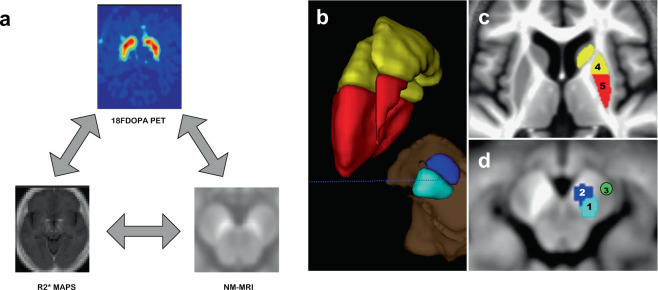
Analyses design and definition of substantia nigra and striatum masks. **a** Diagram showing the three types of relationship explored in this study, between NM-MRI, R2* maps, and F-DOPA PET scan. This diagram will be recalled beside every correlation studied. **b** Tridimensional view of left SN and striatum with their divisions. **c** Axial slice at the level of basal ganglia on the mean structural image of the whole population sample (PD + HC), showing striatum delineation. Precommissural striatum is depicted in yellow (4) and postcommissural striatum in red (5). This division is supported by anatomical and functional data^20,87^. **d** Axial slices at the level of midbrain on a NM-MRI, showing substantia nigra delineation, with division in lateral tiers (1, light blue) and medial tiers (2, dark blue), according to neuropathological data^79^. Cerebral peduncle is also delineated (3, green).

F-DOPA PET, NM-sensitive, and iron-sensitive MRI then probe three different molecular aspects of PD pathophysiology, and although changes in each of these parameters were consistently observed in PD (decreased striatal DA synthesis, NM reduction, and iron accumulation in SN), their joint modifications remain hardly known^15^. This knowledge gap is important to fill in because these molecular markers of PD provide critical and complementary information on PD pathophysiology. In particular, the specific association between iron deposits and NM loss deserve to be investigated as it could provide precious information about pathological processes that might not be parallel in PD neurodegeneration. These relations might be obscured by the heterogeneous subnuclear distribution of dopaminergic neurons in the SN: nearly half of them are packed in discrete zones. The larger of these packs is located in the dorsal and lateral regions of SN^18^, where neuronal loss predominates^19^. Likewise, the depletion of dopamine fibers within the putamen is most pronounced in its posterior portions^20^. The main goal of this study was precisely to compare, between PD patients and healthy control (HC) participants, the relationships linking the dopaminergic function as measured in putamen by [^18^F]DOPA PET to NM content and iron load in the SN, using, respectively, NM- and iron-sensitive MRI, taking into account the predominant pattern of disappearance of dopaminergic neurons and fibers, respectively, in lateral SN and posterior putamen. We systematically probed the effect of disease on R2*, NM-sensitive, and [^18^F]DOPA signal, then the voxelwise relationships between (1) NM and R2* in SN, (2) R2* in SN and whole-brain [^18^F]-DOPA influx rate constant (Ki), and (3) NM in SN and whole-brain Ki (Fig.1a).

## Results

Demographic data appear in Table 1. There were no significant differences between HC and PD patients, in terms of age (two-sample *t* test; *t* (44) = −0.12, *p* = 0.90) and gender (*p* = 1).

**Table 1:** Demographic data.

	Parkinson’s disease patients (PD)	Healthy controls (HC)	Statistic
Total number (*n*)	23	23	
Gender (male:female)	13:10	11:12	Chi-Square: df = 1; value = 0.3485; *p* = 0.5550
Age (years)	67.3 ± 9.9	67.7 ± 9.7	Two-sample *t* test; *t* (44) = −0.12, *p* = 0.90
Most affected side (right:left)	16:7	NA	
Disease duration (years)	5.6 ± 3.8 [1–15]	NA	
Hoehn and Yahr score	1.9 ± 0.6 [1–3]	NA	
UPDRS III (motor)	28.2 ± 11.8 [10–54]	NA	
LEDD (mg)	448.2 ± 286.8 [0–937]	NA	

Values represent the mean ± standard deviation [range] for disease duration, UPDRS III, Hoehn and Yahr score, and LEDD.

*UPDRS* unified Parkinson’s disease rating score^71^, *LEDD* levodopa equivalent daily dose^73^.

**Fig. 2:**
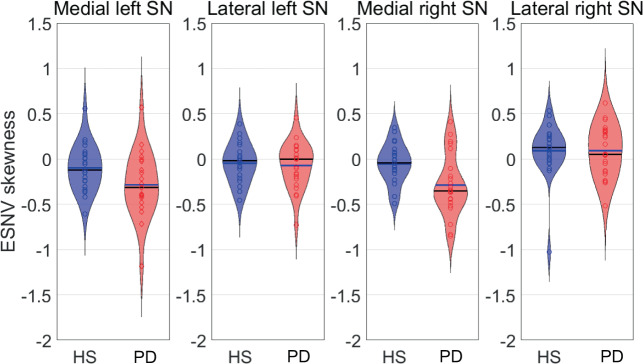
Effective substantia nigra values (ESNV) skewness violin plot. ESNV skewness distribution in HC and PD patients in the four SN subregions (PD patients are depicted in red and HC in blue).

Effective SN values (ESNV, see “Methods” section) skewness was normally distributed across participants (Kolmogorov–Smirnoff, *p* = 0.124). We observed a significant effect of group (*F*(1) = 7.10; *p* = 0.011) and SN ROIs (*F*(3) = 9.76; *p* < 0.001). ESNV skewness was more negative in PD than in HC (least square estimates: −0.138 in PD, −0.02 in HC; *t* (44) = 2.66, *p* = 0.011, Tukey adjustment for multiple comparison; Fig. 2). By contrast, there was not any significant group by ROI interaction (*F*(3) = 1.95, *p* = 0.125). We considered that the decrease in ESNV skewness quantitates SN depigmentation. These results confirm the depopulation of NM-containing neurons in PD, as PD individuals have more voxels with low NM signal than voxels with high NM signal (see Supplementary Fig. 1). They also suggest that the depigmentation evenly involves all SN subdivisions. Finally, no correlation was found between ESNV skewness and unified Parkinson’s disease rating scale part III (UPDRS III), disease duration, Hoehn and Yahr (H&Y) score, or levodopa equivalent daily dose (LEDD).

**Fig. 3:**
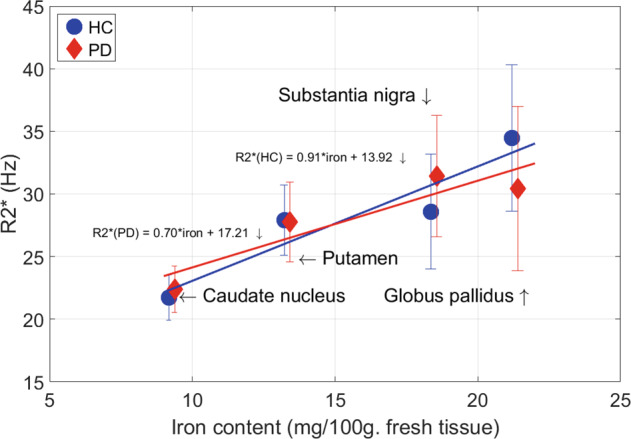
Correlation between published regional brain iron content and regional R2* values of the present study. R2* values extracted from our iron-sensitive MRI data were related to regional brain iron content measured from non-PD post mortem brains, as published by Hallgren and Sourander^21^.

We first confirmed that R2* values were related to regional brain iron content extracted from non-PD post mortem material published in the landmark study by Hallgren and Sourander^21^, a quality control suggested by Martin et al.^22^ (Fig. 3).

**Fig. 4:**
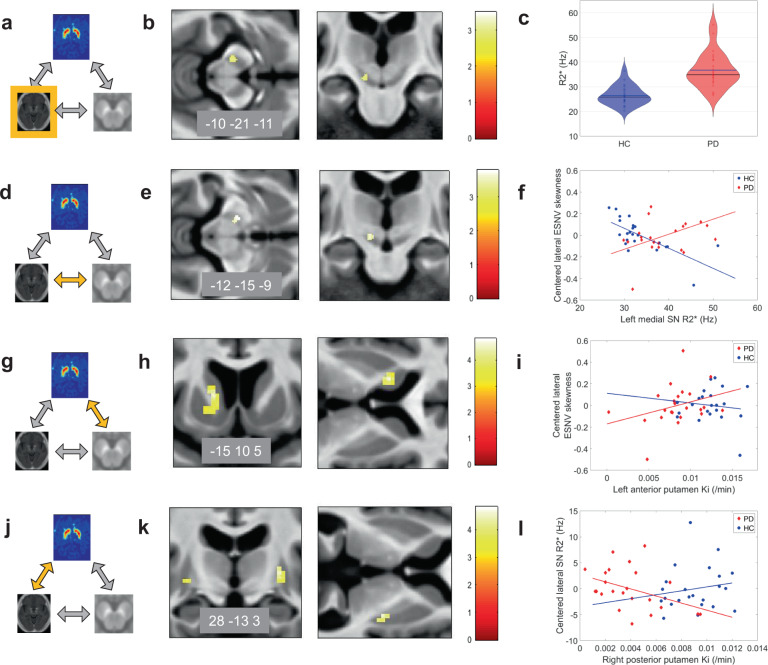
Relationships between NM-sensitive MRI, R2*, and [18F]DOPA PET changes in Parkinson’s disease. **a**–**c** R2* changes in Parkinson’s disease. Main effect of disease on brain iron, as assessed by R2*, displayed at *p* < 0.001 uncorrected over the mean structural image of the whole population. Color scales are proportional to *t* values. **d**–**f** Relationship between R2* and NM-sensitive MRI data. **f** Peak voxel ESNV skewness in both SN increases as R2* increases in left SN, significantly more in PD patients than HC. **g**–**i** Relationship between dopaminergic neurotransmission ([18F]DOPA PET) and NM-sensitive MRI data. Regression between peak voxel in left anterior putamen Ki and bilateral SN ESNV skewness. **j**–**l** Relationship between dopaminergic neurotransmission ([18F]DOPA PET) and R2*. Peak voxel Ki values in posterior putamen regressed differentially in PD and HC with lateral SN R2*. All results are displayed at *p* < 0.001 uncorrected over the mean structural image of the whole population. Color scales are proportional to *t* values.

We then looked for the voxelwise main effect of disease on brain R2*. Two-sample *t* tests revealed that R2* in the left SN lateral tier was significantly higher in PD patients than in controls ([−10 −21 −11] mm; *Z* = 3.12; *p*_SVC_ = 0.041; the result does not survive correction for multiple comparisons across two ROIs—medial and lateral SN) suggesting a higher iron content in that area. The lateralization of R2* increase is consistent with the clinically most affected side, as data were all positioned according to the latter (Fig. 4a–c).

By contrast, we did not find any significant variation of R2* according to clinical parameters (UPDRS III, H&Y scale, disease duration, or LEDD).

We then wondered whether brain R2* would covary with lateral SN ESNV skewness, looking for a relationship between iron accumulation and SN pigmented neurons loss. In a dorsal part of left medial SN, we observed a significant difference between PD patients and HC in the regression between R2* and SN ESNV skewness (group by ESNV skewness interaction; [−12 −15 −9 mm]; *Z* = 3.50; *p*_SVC (medSN)_ = 0.011). However, as shown on Fig. 4d–f, ESNV skewness in lateral SN unexpectedly increases significantly as R2* increases in left medial SN in PD patients, a behavior which is not observed in HC. This result suggests that lateral SN ESNV skewness becomes more positive, as iron accumulation progresses in the medial SN or, alternatively that lateral ESNV skewness decrease (i.e., depigmentation) is associated with decreased iron content in medial SN.

**Fig. 5:**
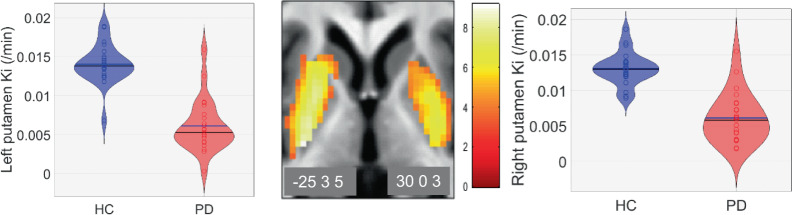
Changes in dopaminergic neurotransmission. Effect of disease on dopaminergic function measured by [^18^F]DOPA PET. Results are displayed at *p* < 0.001 uncorrected over the mean structural image of the whole population. Color scales are proportional to *t* values.

We checked the effect of disease on the function of nigro-striate neurons, as assessed by in vivo measure of dopamine synthesis (Ki). As expected, putamen Ki values were significantly lower in PD patients than in HCs on both sides ([−25 3 5] mm, *Z* = 6.46, *p*_FWE_ < 0.001; [30 0 3], *Z* = 5.54; *p*_FWE_ < 0.001, over the whole brain) (Fig. 5), thus representing the loss of dopaminergic terminals.

We further assessed whether there was any relationship between dopaminergic function and NM-sensitive MRI data. We found a significant between-group difference in the regression between Ki and bilateral SN ESNV skewness in the left anterior putamen (group by ESNV skewness interaction; [−15 10 5] mm, *Z* = 4.57, *p*_SVC_ = 0.002; Fig. 4g–i). In PD, left anterior putamen, oddly enough not the most denervated part of the striatum^20^, Ki significantly decreased as individual lateral SN ESNV skewness decreased, whereas no significant regression was found in HC. This result suggests that, in PD, dopamine synthesis is increasingly impaired as SN depigmentation, labeled by a more negative skewness of ESNV distribution, progresses (Supplementary Fig. 1).

Eventually, we wondered whether dopaminergic function varies in proportion to SN iron accumulation, as indicated by R2* measures. [^18^F]DOPA Ki values of bilateral posterior putamina regressed more tightly in PD than HC with SN R2* of lateral SN, significantly only in the right side ([28 −13 3] mm, *Z* = 4.14, *p*_SVC_ = 0.005), indicating that putamen denervation progresses as iron accumulation (R2*) increases in lateral SN (Fig. 4j–l). This regression was significant in PD ([28 −13 3] mm, *Z* = 3.76, *p*_FWE_ = 0.024) but not in HC. In addition, these differences differed between posterior and anterior putamen, although the triple interaction (group × R2* × ROI) did not survive correction for multiple comparisons.

## Discussion

In this study, three separate imaging biomarkers were, for the first time, jointly used to characterize basal ganglia structure and function in PD patients, as compared to HC: R2* iron-sensitive and NM-sensitive MRI and [^18^F]DOPA PET. The key results are fivefold: As compared to HC, (1) dopamine accumulation in presynaptic terminals is decreased in PD striatum; (2) NM depigmentation does not progress differently across SN subdivisions and it results in an augmented amount of low NM signal voxels, i.e., a more negatively skewed distribution; (3) by contrast, iron selectively tends to accumulate in the lateral SN of PD patients, as shown by the hint of a larger R2* in left lateral SN in PD than HC; (4) dopaminergic function decreases in anterior putamen as the distribution of NM-sensitive signal is negatively skewed by PD, indicating that the impairment of dopaminergic neurons progresses with lateral SN depigmentation; and (5) dopaminergic function negatively relates to lateral SN R2* in PD, suggesting that dopaminergic function deteriorates as iron accumulates in lateral SN. Unexpectedly, R2* in medial SN differs significantly between PD and HC as lateral SN ESNV skewness increases. This suggests that although both signals in the lateral SN covary with dopaminergic function, their mutual interactions are not straightforward. Indeed, this finding suggests that iron accumulates in the medial SN as high NM signal voxels are over-represented therein.

The signal of NM-sensitive MRI in a neuropathological study of three brains (one PD) was found to correspond to the density of residual NM-containing neurons^14^. Importantly, the signal was not influenced by iron deposition. Likewise, iron complexed in NM does not profoundly modify NM-sensitive MRI signal^23^. Several studies showed a reduction of NM-MRI contrast in the SN of PD patients (^15,24,25^ but see ref. ^26^ for negative results), over and above an age-dependent SN depigmentation^27^. However, current NM-sensitive MRI is, at best, semiquantitative. NM SN signal, extracted manually or semiautomatically, has to be corrected for background signal. The latter is taken from either crus cerebri^16,28–30^, pontine tegmentum^31^, or superior cerebellar peduncles^32^. The ensuing dependent variable is either a contrast-to-noise ratio^30,33^ or the number of voxels above an experimenter-defined threshold value^29,31,34^. Here, we resorted to a data-based, reproducible, and fully automatic extraction of SN values from SN voxels identified on an independent probabilistic atlas of basal ganglia. Voxel values in SN were generated simply as the difference between SN and background signal. Individual ESNV were not distributed normally, we thus derived their third moment which allowed us to depict their distribution by a single quantitative value. ESNV skewness differed between groups, with PD distribution being more negatively skewed than in HC. However, the effect of PD did not predominate in any SN subregion. These results suggest that depigmentation increases the abundance of voxels with low NM density, explaining a decrease in ESNV skewness.

As for iron within the SN of PD, it was found increased by neuropathological studies (^35–37^ but see ref. ^38^ for negative findings). Human local brain iron content can be assessed in vivo by qualitative (susceptibility-weighted imaging)^29,39^ or quantitative MRI techniques (R2*, R2^40^, susceptibility mapping^41^). Accordingly, a number of studies^22,42–45^ and two meta-analysis^17,46^ report an increase in iron in the SN of PD patients, although negative findings were also reported^30^. Our results concord with the former results and detect an increase of R2* in PD left lateral SN.

R2* signal is primarily modulated by local iron content, although myelin and calcium content, fiber orientation relative to the magnetic field, and macroscopic geometry also participate in T2* signal decay^47^. Nevertheless, we confirmed that R2* predicts regional brain iron content, as measured by biochemical methods on non-PD human brain samples^21^ (Fig. 3). The effect size (R^2^ = 0.82) indicates that iron explains a major part of R2* signal in basal ganglia. Most of brain iron is bound to ferritin^48^, which is more abundant in glial cells than in neurons^49^. Within SN dopaminergic neurons, iron is primarily stored within NM, which boasts antioxidant and radical scavenging properties contributing to buffer cellular oxidative stress by binding free iron^50,51^. Dopaminergic neurons would selectively degenerate^5^ when NM antioxidant properties are exceeded^52^. At neuronal death, NM is released in the extracellular space where it stimulates inflammation. As a consequence, in PD SN, iron is observed mainly in reactive microglia rather than in dopaminergic NM-positive neurons^11,53^.

The topography of iron deposition in SN is still a matter of debate: some authors found R2* increase in lateral^17^ or posterior SN^54^, while others report an augmented R2* in medial^42^ or anterior SN^55^. We here observed the increase in R2* in the lateral SN of PD patients and postulate that it mainly reflects the accumulation of iron in microglia, as a result of the neuroinflammation accompanying the neuronal loss.

[^18^F]DOPA uptake rate constants correlate with dopamine cells counts^10^. However, [^18^F]DOPA PET, being a functional biomarker, possibly underestimates the loss in dopaminergic neuron because of a compensatory upregulation of amino-acid decarboxylase activity in remaining cells^30,56,57^. Nonetheless, we found that [^18^F]DOPA PET signal is related to microstructural information conveyed by iron- and NM-sensitive MRI data.

An original finding consisted of the relationship between putamen Ki and NM-sensitive data in SN. It illustrates the deterioration of dopaminergic function as depigmentation progresses in the SN of PD patients. This result is supported by similar studies, which resorted to dopamine transporter (DAT) imaging to validate NM-sensitive MRI^30,32,58,59^. In consequence, NM-sensitive MRI is often presented as a useful tool to assess Parkinson’s disease pathology as it can predict dopaminergic dysfunction. Unexpectedly, the alteration of dopaminergic function related to SN depigmentation predominates in the anterior putamen, where dopamine levels decrease less than in the posterior putamen^20^. This finding suggests that depigmentation is an early event, which results in a proportional decrease in dopamine secretion. By contrast, this relationship is lost with more advanced deterioration of nigro-striate function.

We found that in PD, SN R2* increases as Ki decreases in posterior putamen. This result suggests that iron accumulation in lateral SN is a late process which follows and concurs to^60^ the deterioration of dopaminergic function in posterior putamen, the area where dopamine depletion predominates.

[^18^F]DOPA PET and DAT SPECT reflect two different aspects of dopaminergic neurotransmission. The former is directly representative of dopamine synthesis which is the core molecular defect of PD^57^. [^18^F]DOPA PET still represents a robust validated^10^ biomarker for its ability to detect and monitor dopamine deficiency, disease progression^11^, and for its correlation with clinical rating^12,61,62^. By contrast, DAT imaging probes the re-uptake sites of dopaminergic synapses. It does not predict the number of nigral neurons in Parkinson disease^63^ but would be less prone to underestimate dopaminergic dysfunction, as compared to [^18^F]DOPA PET^57^.

As for the relations between iron- and NM-sensitive MRI data, we found that medial SN R2* is proportional to lateral SN ESNV skewness, significantly more in PD than in HC. A first explanation would posit that iron accumulation in medial SN decreases as low NM signal voxels accumulate in PD, suggesting that neuroinflammation decreases in medial SN as depigmentation progresses in lateral SN. This interpretation is counterintuitive but might emphasize a regionally specific time course of iron accumulation within SN in PD. Alternatively, this result would arise fortuitously from our limited sample of PD patients. Indeed, relationships between R2* and NM signal are known to be weak^44^.

This study boasts some methodological strengths. First, most studies exploring the brain correlates of PD used manually drawn regions of interest^11,64,65^. Such methods are prone to observer bias and cannot be easily compared between laboratories. By contrast, we extracted regions of interest from an independent atlas, freely available to the community. Second, in order to observe the effect of disease processes properly, we strictly matched for age PD patients with HC, on an individual basis. Indeed, brain iron levels increase as a function of age in deep gray matter (GM) structure^21,51,66^ and age-dependent changes were to be accounted for.

The study also presents some limitations, the first one being the small number of participants. Considering the duration of MRI and PET acquisitions, we could not include patients with advanced PD (H&Y scale > 3) given the discomfort of the experiment. In consequence, the results only show effects predominant in stages H&Y 1–3 or effects stable over for a long period of time. We did not recruit patients with atypical parkinsonism, which did not allow us to study the specificity of the results for the identification of PD and its differentiation from other akinetic-rigid syndromes. Another limitation comes from the methodological sophistication of a group analysis which is not available in most hospitals, thereby of little help for individual PD diagnosis in clinical practice.

We did not find any correlation between imaging data and clinical scores, in concordance with previous reports^32,67^. Most of the time, results are mixed. In Schwarz et al.’s^34^ study for example, there was no significant correlation between disease duration or H&Y and NM signal intensity, whereas a correlation with UPDRS was found^34^.

We have explored the role of these neuroimaging techniques as diagnostic biomarkers. Such biomarkers ideally quantify the presence of the disease, and can be used to provide new insights into pathogenic pathways^68^. [18F]DOPA PET does not measure the number of dopaminergic neurons, but rather measure a biologic process. NM-MRI offers the opportunity to measure the amount of surviving pigmented neurons. Iron-sensitive MRI shows another aspect of the neuropathological process through iron deposits measurements. The specificity of these two neuroimaging biomarkers regarding PD diagnosis remains to be investigated as our population did not include patients suffering from atypical parkinsonism. While it was proven that there is a progressive regional decline in striatal [18F]DOPA uptake with PD evolution^69,70^, we cannot make any statement about the use of NM-MRI and iron-sensitive MRI as biomarkers of disease progression as we did not study the alteration of the signal over time.

Finally, this cross-sectional study cannot characterize the evolution of these MRI biomarkers along time, nor specify the differential course of iron, NM, and [^18^F]DOPA signals, as they might label different pathological processes.

In conclusion, our results show that (1) iron selectively increases in lateral SN; (2) iron in lateral SN increases as dopaminergic function fails in posterior putamen; (3) depigmentation of SN does not show any regional specificity within SN and correlates with dopaminergic failure in anterior putamen; and (4) despite their respective association with dopaminergic function, iron and NM signals show regionally complex interactions. These metrics convey information about separate processes, respectively, inflammation and loss of NM-containing neurons, which might unfold following different time courses. Our study speaks for (1) the need to develop MRI sequences quantifying NM and (2) longitudinal studies of iron- and NM-sensitive imaging to assess their respective sensitivity and specificity to the natural individual evolution of PD across time.

## Methods

### Patients

Forty-six participants took part in this study, which was approved by the local ethic committee (Belgian approval number 2012/79), and was performed in accordance with the ethical standards described in the Declaration of Helsinki (1964). Written informed consent was obtained from all participants. Twenty-three patients were recruited at the movement disorder clinic of the CHU Liège, Belgium, with a diagnosis of PD according to UK Brain Bank Criteria^8^ and Movement Disorders Society guidelines^6^, excluding atypical parkinsonism, vascular and other secondary parkinsonisms. Six patients had early parkinsonism (disease duration ≤ 2 years) and the diagnosis was confirmed 4 years later, using the same criteria (UK Brain Bank and MDS guidelines). All patients had a positive response to dopaminergic drugs/agents. The inclusion criteria were (1) age between 40 and 90 years, (2) H&Y scale < 4, (3) compatibility with MRI, and (4) no pregnancy. Twenty-three HC participants individually matched for age and gender, free from neurological or psychiatric disease, followed the exact same experimental protocol.

### Design overview

Participants were invited on a single day to MRI and [^18^F] DOPA PET imaging sessions. MRI and PET sessions were respectively scheduled in the morning and the afternoon. Clinical assessments were interleaved between imaging sessions.

Parkinson’s disease patients were assessed with UPDRS III^71^, by two experienced raters to determine motor severity in OFF state (which was defined as absence of any treatment (*n* = 3) or treatment discontinuation for at least 24 h for all the others). Clinical laterality was established based on medical history and examination at the Movement Disorder Unit. The disease stage was determined using the H&Y scale. Disease duration was estimated as the period since first recalled motor symptoms^72^. LEDD was calculated for each patient^73^.

### MR image acquisition, preprocessing, and PET acquisitions

**Fig. 6:**
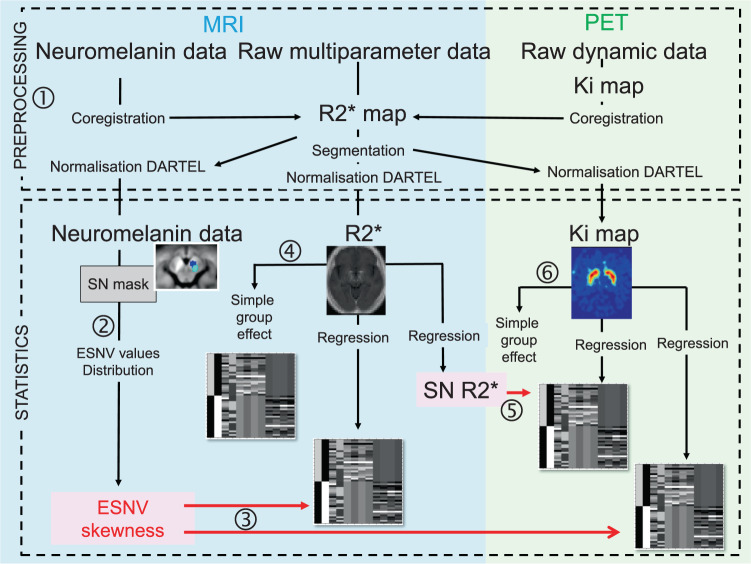
Chartflow of data processing and analyses (see text). 1. Preprocessing ensures that MRI and PET data are finally registered in a common space. 2. Neuromelanin data consist of ESNV across all SN voxels, corrected for baseline values taken from bilateral cruces. 3. ESNV skewness act as regressor for R2* and Ki maps. 4. R2* maps serve in a group comparison. 5. R2* values in SN serve as regressor for Ki maps. 6. Ki map are used to show the voxelwise group effect. The gray diagrams illustrate design matrices.

All scans were conducted at the GIGA Cyclotron Research Centre-In Vivo Imaging Laboratory of the University of Liège, Belgium. MRI data were acquired on a 3T whole-body MRI-scanner (Magnetom Prisma, Siemens Medical Solutions, Erlangen, Germany). Chartflow of image analysis appear on Fig. 6.

Whole-brain MRI acquisitions included a multiparameter mapping (MPM) protocol^74,75^ that allows voxelwise R2* quantification (as well as magnetization transfer (MT) saturation, R1 and PD estimation). The whole-brain MRI acquisitions included a MPM protocol, developed in the framework of an international collaborative effort (Weiskopf et al.^74^; Tabelow et al.^75^). This protocol consists of three colocalized 3D multiecho fast low angle shot (FLASH) acquisitions at 1 × 1 × 1 mm^3^ resolution and two additional calibration sequences to correct for inhomogeneities in the RF transmit field^76,77^. The FLASH data sets were acquired with predominantly proton density (PD), T1, and MT weighting, referred to in the following as PDw, T1w, and MTw, acquired at different echo times. All three had high bandwidth to minimize off-resonance and chemical shift artifacts. Volumes were acquired in 176 sagittal slices using a 256 × 224 voxel matrix. GRAPPA parallel imaging was combined with partial Fourier acquisition to speed up acquisition time to ~20 min.

NM-sensitive (NM-MRI) images of the brainstem were recorded, using high-resolution 3D-FLASH sequence including an MT preparation pulse for NM sensitization^78^. The following acquisition parameters were used: FoV = 256 × 232 mm^2^, 52 slices (+23% oversampling), matrix size = 256 × 232 × 52, 1 mm isotropic resolution, TR = 30 ms, TE = 2.61 ms, flip angle = 23°, GRAPPA acceleration factor 2, 6/8 partial Fourier in through-plane direction, 3 averages, bandwidth = 450 Hz/pixel, and acquisition time = 9′10″. The TR was made longer (30–35 ms) to fulfill SAR limitations when needed (normal SAR operating mode for all patients). A T1-weighted anatomical image [3D magnetization-prepared rapid gradient echo sequence] acquired in the same session was used to position the slices accurately.

All participants received 100 mg of oral carbidopa (Lodosyn^®^ 25 mg) 1 h before tracer injection and 50 mg at injection time. Patients were asked to interrupt their treatment at least 12 h before the experiment (withdrawal of L-dopa, rasagiline, entacapone, amantadine, dopamine agonist, and 48 h discontinuation for prolonged duration formulations).

PET imaging was conducted on an ECAT EXACT HR+ scanner (Siemens CTI, Knoxville, TN). Head movements were minimized using a thermoplastic mask molded on the subject’s head. After a 10-min transmission scan for attenuation correction, participants received a single dose of [^18^F]-DOPA administered as a 1-min bolus in an antecubital vein (injected dose 313.78 ± 20.25 SD MBq). The dynamic image acquisition started immediately after injection and consisted of 34 frames of 6 × 10, 8 × 30, 5 × 120, and 15 × 300 s (total = 90 min). All PET images were reconstructed using filtered back projection (Hann filter, 4.9 mm FWHM) including corrections for attenuation, dead time, random and scatter events using standard software (ECAT 7.1, Siemens/CTI, Knoxville, TN). With these acquisition and reconstruction settings, the transaxial resolution in water is 6.5 mm in the brain volume (voxel size 2.57 × 2.57 × 2.43 mm^3^).

### Image processing

We had strong predictions about where significant changes would be observed: the SN and the striatum. Based on the literature and neuropathological data^79^, we further split SN into lateral and medial tiers on each side (Fig. 1b, regions 1 and 2; with volumes of left medial, right medial, left lateral, and right lateral SN of 164, 130, 137, and 100 mm^3^). Likewise, striatum was divided into precommissural, postcommissural, left, and right striatum (Fig. 1b, regions 4 and 5^20,80^; with volumes of left anterior putamen, right anterior putamen, left posterior putamen, and right posterior putamen of 5356, 3787, 5322, and 3203 mm^3^). ATAG atlas of elderly population striatum and SN (freely available on https://www.nitrc.org/projects/atag) has been nonlinearly registered to DARTEL study space^81^. Binary masks were derived from the probabilistic images, using an experimenter-defined threshold [corresponding to 4 a.u. (according to ATAG metrics)] that ensures optimal anatomical coverage of SN and striatum. To generate regressors for statistical analyses, SN masks were used to extract values of SN voxels. Data from NM- and iron-sensitive sequences were extracted separately on each side from medial and lateral SN, keeping in mind that NM signal loss is known to predominate in lateral SN^15,33^. By contrast, for statistical inferences, we used either bilateral masks of medial and lateral SN (for the regression between R2* maps and NM signal) or pre- and postcommissural striatum (for regression with Ki maps).

Masks for bilateral cruces cerebri consisted of two 14-mm^3^ spheres positioned by the experimenter on the mean study structural image (Fig. 1b, region 3). These masks were used to compute the background signal for SN values (see below). Finally, for voxelwise statistics, an explicit GM mask was used as previously proposed^82^: the smooth modulated warped individual GM and white matter (WM) maps were averaged across all subjects and the GM mask only included voxels for which the mean GM probability was (1) larger than that of WM and (2) above 20%. This ensures that the GM mask includes voxels (on average over the population) with a sufficient amount of GM and more than of WM.

R2* maps were processed using Matlab R2015b (MathWorks Inc., Natick, MA, USA), SPM12 (Welcome Trust Centre for Neuroimaging, London, UK; www.fil.ion.ucl.ac.uk/spm), and a dedicated SPM toolbox for quantitative MRI (hMRI toolbox, http://hmri.info^75^). Quantitative maps of PD patients and controls were segmented using the “unified segmentation” scheme^81^. GM and WM probability maps from all subjects were then warped together into a study-specific reference space, based on diffeomorphic transformations (DARTEL), and aligned with the MNI space, providing a subject-specific deformation field^83^. For voxel-based analyses, R2* maps were normalized using the subject-specific deformation field without modulation. A tissue-weighted smoothing (4 mm FWHM isotropic) yielded a smoothed tissue-specific multiparameter map which optimally preserved quantitative parameter values within each tissue class^75,84^. Parametric maps of seven PD patients were flipped according to the clinically most impaired side (the latter was identified following clinical examination by two experienced movement disorder specialists, taking into account the highest UDPRS score of each side), in order to gather the most affected sides of all patients on the left side of the images. However, it must be kept in mind that many of our patients had already bilateral symptoms and signs.

For NM-sensitive MRI, voxel values included in the SN and crus masks were extracted for each participant. NM content was parametrized simply as the difference between any given SN voxel and background values estimated as the median signal in crus cerebri and will be referred to as “ESNV” [ESNV = S_i,SN_ − mean (S_crus_), with S_i,SN_, the value of the *i*th voxel; median (S_crus_), the median value of crus]. Because individual ESNV distributions were not normal (Kolmogorov–Smirnov; *p* = 0.0157), we derived their skewness, which ultimately served as the dependent variable because it was shown sensitive to SN depigmentation (see “Results”).

PET data were preprocessed using SPM12. For each participant, an average PET image was created using the data acquired between 8 and 20 min corresponding to frames 15–19. The count statistics in this average image makes it a better source image for coregistration of PET data to structural MRI. The average PET image was coregistered to the individual structural MRI and normalized in the common standard space using the flow-field deformation parameters obtained during the spatial normalization of structural MRI. Two regions of interest (putamen and occipital cortex), with and without specific uptake respectively, were identified using the Automated Anatomical Labelling (AAL) atlas^85^. These two regions were transferred to the space of individual dynamic PET series, after applying the inverse normalization transformations, then used to extract time–activity curves needed to compute parametric maps of [^18^F]-DOPA influx rate constant (Ki). In order to avoid any additional partial volume effect which might be introduced during the preprocessing of the data, we preferred to send the AAL ROIs back into the subject space, extract the time series directly from the raw data, and then create the Ki maps in the subject space. Kinetic modeling using dynamic PET data and time–activity curves for reference region (occipital cortex and putamen) was conducted in PMOD software (version 3.7, PMOD Technologies, Zurich, Switzerland). Patlak linear graphical analysis was used to compute brain parametric images of Ki map^86^. The time–activity curve in putamen was used to determine the start time of the linear segment (t*) of the graph (estimated to 30 min) and used to compute voxelwise Ki. The Ki map, generated in the subject space, was then normalized into the MNI space by applying individual normalization parameters computed on the average PET image. Finally, images were smoothed with an isotropic Gaussian kernel of 4 mm.

### Statistical analyses

Voxelwise one-tailed “two-sample *t* test” assessed the group effect by comparing Ki between healthy subjects and PD patients.

A multiple regression tested for the interaction between the group effect and SN R2*. Median R2* was derived from lateral, medial, left, and right SN, centered and included as group specific regressors in an analysis, which also included age and sex as regressors of no interest. Inferences were conducted as above. Contrasts tested the regression of bilateral, lateral, and medial, ESNV on Ki maps (Supplementary Table 1).

A last multiple regression tested for the interaction between the group effect and ESNV skewness, as described above for R2* analyses. Contrasts tested the regression of bilateral, lateral, and medial, R2* on Ki maps (Supplementary Table 1). Inferences were conducted as above.

Whole-brain voxel-based quantification analyses relied on multiple linear regression models embedded in the general linear model framework of SPM12.

Statistics on R2* differences between PD patients and HC in GM were estimated by separate ‘two-sample one-tailed *t* tests’. Age and sex were included as regressors of no interest.

Within the patient population, multiple regressions looked for significant voxelwise regression between R2* in GM and clinical scores (UPDRS III in OFF state, H&Y scale, LEDD, disease duration).

Finally, a multiple regression tested voxelwise for the interaction between-group effect and ESNV skewness on R2*. As described above, individual ESNV skewnesses were derived from lateral and medial, left and right SN (see section “Image processing”), centered and included as group specific regressors in the analysis, which also included age and sex as regressors of no interest. Contrasts tested the regression of bilateral, lateral, and medial, ESNV on R2*maps (Supplementary Table 1). In all cases, inferences were conducted at the voxel level at *p* < 0.05 FWE, after small volume correction using the bilateral masks described above. We controlled for the potential type I error over the two masks used for inferences, respectively, for SN (medial and lateral) or striatum (pre and post commissural).

After checking for the normality of ESNV skewness distribution, a generalized linear mixed model tested the effects of group (PD versus HC), SN ROIs (4 sub-ROIs), and their interaction on ESNV skewness, using patient intercept as a random variable and SAS software, version 9.4 (SAS Institute Inc., 2013). The regression between ESNV skewness and UPDRS III, disease duration, H&Y score, or LEDD was also tested.

Statistical analyses on demographic data were computed using SAS software, version 9.4 (SAS Institute Inc., 2013).

### Reporting summary

Further information on research design is available in the Nature Research Reporting Summary linked to this article.

## Supplementary information

Supplementary Information

Reporting Summary

## Data Availability

MRI data supporting the results of this study are available from the corresponding author, on a collaborative basis.

## Code availability

Matlab code developed for this study is available from the corresponding author, on a collaborative basis.
