# Supplementary material for: Parkinson’s disease multimodal imaging: F-DOPA PET, neuromelanin-sensitive and quantitative iron-sensitive MRI
Source: NPJ Parkinsons Dis. 2021 Jul 8;7:57. doi: 10.1038/s41531-021-00199-2 (PMC8266835; doi:10.1038/s41531-021-00199-2)
Supplement: Supplementary file 1 — Supplementary Information [file 41531_2021_199_MOESM1_ESM.pdf]

Supplementary Table 1. Definition of regressor used in the analysis

MAP	REGRESSOR
Left medial SN R2*	Bilateral lateral ESNV
Left anterior putamen Ki	Bilateral lateral ESNV
Bilateral posterior putamen Ki	Bilateral lateral R2*

ESNV (resp. R2*) were extracted from different SN or putamen sub-regions and used as regressors in multiple regression analyses. Contrasts always involved both sides of each subregion and masks used for inference were also bilateral.

Supplementary Figure 1. ESNV distribution.

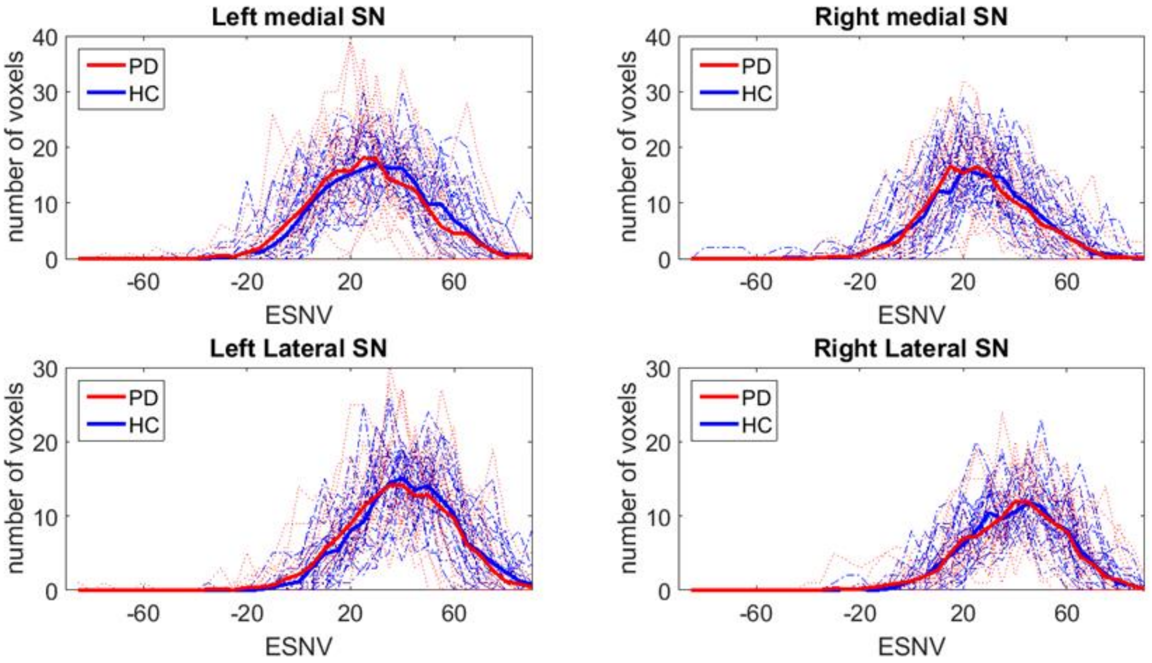

Diagrams show ESNV distribution across PD and HC populations, in all four subregions of SN (PD patients are depicted in red, and HC in blue). ESNV in PD are characterized by a larger tail of small ESNV (i.e., a more negative skewness than in HC, particularly visible on the left side).
