# Supplementary material for: Parkinson’s disease multimodal imaging: F-DOPA PET, neuromelanin-sensitive and quantitative iron-sensitive MRI
Source: NPJ Parkinsons Dis. 2021 Jul 8;7:57. doi: 10.1038/s41531-021-00199-2 (PMC8266835; doi:10.1038/s41531-021-00199-2)
Supplement: Supplementary file 2 — Reporting Summary [file 41531_2021_199_MOESM2_ESM.pdf]

nature research

Corresponding author(s): Depierreux Frederique (NPJPARKD-00646)

Last updated by author(s): Jun 1, 2021

# Reporting Summary

Nature Research wishes to improve the reproducibility of the work that we publish. This form provides structure for consistency and transparency in reporting. For further information on Nature Research policies, see our Editorial Policies and the Editorial Policy Checklist.

## Statistics

For all statistical analyses, confirm that the following items are present in the figure legend, table legend, main text, or Methods section.

| n/a | Confirmed | |
|---|---|---|
| ☐ | ☒ | The exact sample size ($n$) for each experimental group/condition, given as a discrete number and unit of measurement |
| ☒ | ☐ | A statement on whether measurements were taken from distinct samples or whether the same sample was measured repeatedly |
| ☐ | ☒ | The statistical test(s) used AND whether they are one- or two-sided<br>*Only common tests should be described solely by name; describe more complex techniques in the Methods section.* |
| ☐ | ☒ | A description of all covariates tested |
| ☐ | ☒ | A description of any assumptions or corrections, such as tests of normality and adjustment for multiple comparisons |
| ☐ | ☒ | A full description of the statistical parameters including central tendency (e.g. means) or other basic estimates (e.g. regression coefficient) AND variation (e.g. standard deviation) or associated estimates of uncertainty (e.g. confidence intervals) |
| ☐ | ☒ | For null hypothesis testing, the test statistic (e.g. $F$, $t$, $r$) with confidence intervals, effect sizes, degrees of freedom and $P$ value noted<br>*Give P values as exact values whenever suitable.* |
| ☒ | ☐ | For Bayesian analysis, information on the choice of priors and Markov chain Monte Carlo settings |
| ☐ | ☒ | For hierarchical and complex designs, identification of the appropriate level for tests and full reporting of outcomes |
| ☒ | ☐ | Estimates of effect sizes (e.g. Cohen's $d$, Pearson's $r$), indicating how they were calculated |

*Our web collection on statistics for biologists contains articles on many of the points above.*

## Software and code

Policy information about availability of computer code

| | |
|---|---|
| Data collection | No software was used |
| Data analysis | For MRI:<br>Matlab R2015b (The MathWorks Inc., Natick, MA, USA) , SPM12 (Welcome Trust Centre for Neuroimaging, London, UK; www.fil.ion.ucl.ac.uk/spm) and a dedicated SPM toolbox for quantitative MRI (hMRI toolbox, http://hmri.info,25).<br>For PET<br>SPM12 and PMOD software (Version 3.7, PMOD Technologies, Zurich, Switzerland) |

For manuscripts utilizing custom algorithms or software that are central to the research but not yet described in published literature, software must be made available to editors and reviewers. We strongly encourage code deposition in a community repository (e.g. GitHub). See the Nature Research guidelines for submitting code & software for further information.

## Data

Policy information about availability of data

All manuscripts must include a data availability statement. This statement should provide the following information, where applicable:

- Accession codes, unique identifiers, or web links for publicly available datasets
- A list of figures that have associated raw data
- A description of any restrictions on data availability

MR data supporting the results of this study are available from the corresponding author, on a collaborative basis. Data are available on GIGA CRC in vivo imaging server.

# Field-specific reporting

Please select the one below that is the best fit for your research. If you are not sure, read the appropriate sections before making your selection.

☒ Life sciences ☐ Behavioural & social sciences ☐ Ecological, evolutionary & environmental sciences

For a reference copy of the document with all sections, see nature.com/documents/nr-reporting-summary-flat.pdf

# Life sciences study design

All studies must disclose on these points even when the disclosure is negative.

| | |
|---|---|
| Sample size | Power analysis was based on results available for [18F]DOPA PET because no previous data were available for multiparameter protocol. |
| Data exclusions | Fourteen data sets were dropped out because of incorrect diagnosis (2), incomplete data (7) or movement artifact (5). |
| Replication | Most of our results reproduce published data. The originality of our data set lies in the joint characterization of 3 different imaging modalities. |
| Randomization | All consecutive PD patients of the outpatient clinic for movement disorders were proposed the study if they matched inclusion criteria. |
| Blinding | Not applicable. |

# Reporting for specific materials, systems and methods

We require information from authors about some types of materials, experimental systems and methods used in many studies. Here, indicate whether each material, system or method listed is relevant to your study. If you are not sure if a list item applies to your research, read the appropriate section before selecting a response.

## Materials & experimental systems

| n/a | Involved in the study |
|---|---|
| ☒ | ☐ Antibodies |
| ☒ | ☐ Eukaryotic cell lines |
| ☒ | ☐ Palaeontology and archaeology |
| ☒ | ☐ Animals and other organisms |
| ☐ | ☒ Human research participants |
| ☐ | ☒ Clinical data |
| ☒ | ☐ Dual use research of concern |

## Methods

| n/a | Involved in the study |
|---|---|
| ☒ | ☐ ChIP-seq |
| ☒ | ☐ Flow cytometry |
| ☐ | ☒ MRI-based neuroimaging |

## Human research participants

Policy information about studies involving human research participants

| | |
|---|---|
| Population characteristics | Forty-six participants took part in this study; twenty-three patients were recruited at the movement disorder clinic of the CHU Liège, Belgium, with a diagnosis of PD according to UK Brain Bank Criteria and Movement Disorders Society guidelines, excluding atypical parkinsonism, vascular and other secondary parkinsonisms. Six patients had early parkinsonism (disease duration ≤ two years) and the diagnosis was confirmed four years later, using the same criteria (UK Brain Bank and MDS guidelines). All patients had a positive response to dopaminergic drugs/agents. The inclusion criteria were (1) age between 40 and 90 y., (2) Hoehn and Yahr scale<4, (3) compatibility with MRI, (4) no pregnancy. Twenty-three healthy control (HC) participants individually matched for age and gender, free from neurological or psychiatric disease, followed the exact same experimental protocol. |
| Recruitment | The twenty-three PD patients were recruited at the movement disorder clinic of the CHU Liège, Belgium. Healthy controls were recruited by advertisement locally (inclusion criteria were (1) age between 40 and 90 y., (2) free from neurological or psychiatric disease, (3) compatibility with MRI, (4) no pregnancy. They were individually matched for age with PD patients. |
| Ethics oversight | Ethic committee of the University Hospital (CHU) of Liege, Liege, Belgium (Belgian approval number 2012/79) |

Note that full information on the approval of the study protocol must also be provided in the manuscript.

# Clinical data

Policy information about clinical studies

All manuscripts should comply with the ICMJE guidelines for publication of clinical research and a completed CONSORT checklist must be included with all submissions.

| | |
|---|---|
| Clinical trial registration | Belgian approval number 2012/79 |
| Study protocol | Study protocol is registered by the CHU of Liege Ethic Comittee |
| Data collection | Patients and controls were recruited between October 2012 and May 2015. Data collection was realised between May 2015 and April 2016. All scans were conducted at the GIGA Cyclotron Research Centre-in vivo imaging laboratory of the University of Liège, Belgium. |
| Outcomes | The main goal of this study was to compare, between Parkinson's disease patients and healthy control participants, the relationships linking the dopaminergic function as measured in putamen by [18F]DOPA PET to neuromelanin content and iron load in the substantia nigra, using respectively neuromelanin- and iron-sensitive MRI, taking into account the predominant pattern of disappearance of dopaminergic neurons and fibers, respectively in lateral substantia nigra and posterior putamen. We systematically probed the effect of disease on R2*, NM-sensitive and [18F]DOPA signal, then the voxel wise relationships between<br>(1) NM and R2* in substantia nigra,<br>(2) R2* in substantia and whole brain [18F]-DOPA influx rate constant (Ki)<br>(3) NM in SN and whole brain Ki |

# Magnetic resonance imaging

## Experimental design

| | |
|---|---|
| Design type | Structural MRI |
| Design specifications | Not applicable. |
| Behavioral performance measures | MDS-UPDRS score, LEDD, Hoehn & Yahr stages, disease duration. |

## Acquisition

| | |
|---|---|
| Imaging type(s) | Structural |
| Field strength | 3 Tesla |
| Sequence & imaging parameters | - R2*:<br>Whole-brain MRI acquisitions included a multiparameter mapping (MPM) protocol that allows voxelwise R2* quantification (as well as MT saturation, R1 and PD estimation). The whole-brain MRI acquisitions included a multiparameter mapping (MPM) protocol, developed in the framework of an international collaborative effort (Weiskopf et al., 2013; Tabelow et al., 2019). This protocol consists of three co-localized 3D multi-echo fast low angle shot (FLASH) acquisitions at 1 x 1 x 1 mm³ resolution and two additional calibration sequences to correct for inhomogeneities in the RF transmit field. The FLASH data sets were acquired with predominantly proton density (PD), T1 and magnetization transfer (MT) weighting, referred to in the following as PDw, T1w and MTw, acquired at different echo times. All three had high bandwidth to minimize off-resonance and chemical shift artifacts. Volumes were acquired in 176 sagittal slices using a 256 x 224 voxel matrix. GRAPPA parallel imaging was combined with partial Fourier acquisition to speed up acquisition time to approximately 20 minutes.<br>- NM-sensitive MRI:<br>Neuromelanin-sensitive (NM-MRI) images of the brainstem were recorded, using high resolution 3D-FLASH sequence including an MT preparation pulse for NM-sensitization. The following acquisition parameters were used: FoV = 256 x 232 mm², 52 slices (+23% oversampling), matrix size = 256 x 232 x 52, 1 mm isotropic resolution, TR = 30 ms, TE = 2.61 ms, flip angle = 23°, GRAPPA acceleration factor 2, 6/8 partial Fourier in through-plane direction, 3 averages, bandwidth = 450 Hz/pixel, acquisition time = 9’10”. The TR was made longer (30-35 ms) to fulfill SAR limitations when needed (normal SAR operating mode for all patients). |
| Area of acquisition | - R2*: Whole-brain MRI acquisitions<br>- NM-sensitive MRI: 52 slices placed at the level of midbrain. A T1-weighted anatomical image [3D magnetization-prepared rapid gradient echo (MPRAGE) sequence] acquired in the same session was used to position the slices accurately. |

Diffusion MRI ☐ Used ☒ Not used

## Preprocessing

| | |
|---|---|
| Preprocessing software | R2* maps were processed using Matlab R2015b (The MathWorks Inc., Natick, MA, USA) , SPM12 (Welcome Trust Centre for Neuroimaging, London, UK; www.fil.ion.ucl.ac.uk/spm) and a dedicated SPM toolbox for quantitative MRI (hMRI toolbox, http://hmri.info). Quantitative maps of PD patients and controls were segmented using the ‘unified segmentation’ scheme. GM (grey matter) and WM (white matter) probability maps from all subjects were then warped together into a study- |

| | |
|---|---|
| | specific reference space, based on diffeomorphic transformations (DARTEL) , and aligned with the MNI space, providing a subject-specific deformation field |
| Normalization | For voxel-based analyses, R2* maps were normalized using the subject-specific deformation field without modulation. A tissue-weighted smoothing (4 mm FWHM isotropic) yielded a smoothed tissue-specific multiparameter map which optimally preserved quantitative parameter values within each tissue class. |
| Normalization template | GM (grey matter) and WM (white matter) probability maps from all subjects were then warped together into a study-specific reference space, based on diffeomorphic transformations (DARTEL) , and aligned with the MNI space, providing a subject-specific deformation field |
| Noise and artifact removal | Not applicable. |
| Volume censoring | None. |

## Statistical modeling & inference

| | |
|---|---|
| Model type and settings | Mass univariate multiple regression model based on GLM |
| Effect(s) tested | Group (PD vs HC) and group by SN R2* (resp. NM signal) |

Specify type of analysis: ☐ Whole brain ☐ ROI-based ☒ Both

| | |
|---|---|
| Anatomical location(s) | To generate regressors for statistical analyses, substantia nigra masks were used to extract values of substantia nigra voxels. Data from NM- and iron-sensitive sequences were extracted separately on each side from medial and lateral substantia nigra. Substantia nigra masks come from ATAG atlas of elderly population (freely available on https://www.nitrc.org/projects/atag), which has been non linearly registered to DARTEL study space. |

| | |
|---|---|
| Statistic type for inference (See Eklund et al. 2016) | Amplitude of change at the voxel level. |
| Correction | FWE over prespecified ROIs, independently identified. |

## Models & analysis

| n/a | Involved in the study |
|---|---|
| ☒ | ☐ Functional and/or effective connectivity |
| ☒ | ☐ Graph analysis |
| ☒ | ☐ Multivariate modeling or predictive analysis |
